# Cordycepin Attenuates Testosterone-Induced Benign Prostatic Hyperplasia in Rats via Modulation of AMPK and AKT Activation

**DOI:** 10.3390/pharmaceutics14081652

**Published:** 2022-08-08

**Authors:** Abdulmohsin J. Alamoudi, Sami A. Alessi, Waleed Y. Rizg, Abdulmajeed M. Jali, Awaji Y. Safhi, Fahad Y. Sabei, Sameer Alshehri, Khaled M. Hosny, Ashraf B. Abdel-Naim

**Affiliations:** 1 Department of Pharmacology and Toxicology, Faculty of Pharmacy, King Abdulaziz University, Jeddah 21589, Saudi Arabia; 2 Center of Research Excellence for Drug Research and Pharmaceutical Industries, King Abdulaziz University, Jeddah 21589, Saudi Arabia; 3 Department of Pharmaceutical Care, King Abdulaziz Hospital, Jeddah 21589, Saudi Arabia; 4 Department of Pharmaceutics, Faculty of Pharmacy, King Abdulaziz University, Jeddah 21589, Saudi Arabia; 5 Department of Pharmacology and Toxicology, College of Pharmacy, Jazan University, Jazan 45142, Saudi Arabia; 6 Department of Pharmaceutics, College of Pharmacy, Jazan University, Jazan 45142, Saudi Arabia; 7 Department of Pharmaceutics and Industrial Pharmacy, College of Pharmacy, Taif University, P.O. Box 11099, Taif 21944, Saudi Arabia

**Keywords:** cordycepin, benign prostatic hyperplasia, testosterone, AMPK, AKT

## Abstract

Benign prostatic hyperplasia (BPH) is a disease that commonly affects elderly men. Cordycepin is an adenosine analog with a wide range of pharmacological activities including antiproliferative and prostatic smooth muscle relaxant effects. This study was designed to assess the actions of cordycepin in testosterone-induced BPH in rats. Animals were divided into six treatment groups: control, cordycepin-alone (10 mg/kg), testosterone-alone (3 mg/kg), cordycepin (5 mg/kg) + testosterone, cordycepin (10 mg/kg) + testosterone, and finasteride (0.5 mg/kg) + testosterone. Treatments were continued daily, 5 days a week, for 4 weeks. Cordycepin significantly prevented the increase in prostate weight and prostate index induced by testosterone. This was confirmed by histopathological examinations. Cordycepin antiproliferative activity was further defined by its ability to inhibit cyclin-D1 and proliferating cell nuclear antigen (PCNA) expression. In addition, cordycepin exhibited significant antioxidant properties as proven by the prevention of lipid peroxidation, reduced glutathione diminution, and superoxide dismutase exhaustion. This was paralleled by anti-inflammatory activity as shown by the inhibition of interleukin-6, tumor necrosis factor-α, and nuclear factor-κB expression in prostatic tissues. It also enhanced apoptosis as demonstrated by its ability to enhance and inhibit mRNA expression of Bax and Bcl2, respectively. Western blot analysis indicated that cordycepin augmented phospho-AMP-activated protein kinase (p-AMPK) and inhibited p-AKT expression. Collectively, cordycepin has the ability to prevent testosterone-induced BPH in rats. This is mediated, at least partially, by its antiproliferative, antioxidant, anti-inflammatory, and pro-apoptotic actions in addition to its modulation of AMPK and AKT activation.

## 1. Introduction

Benign prostatic hyperplasia (BPH) is characterized by enhanced multiplication of prostatic stromal and epithelial cells. PBH is a common disease within the elderly male population [1]. According to several reports, the incidence of BPH rises with age [2]. BPH symptoms are thought to affect about half of all men over the age of 60 [3]. Symptoms of BPH can significantly and negatively impact the quality of life of affected men [4]. These symptoms of BPH include decreased urinary flow and urinary incontinence, nocturia, dribbling, and difficulty initiating flow [5]. The pathogenesis of BPH has been linked to oxidative stress, chronic inflammation, and disturbed apoptosis [6,7]. The two main medical treatments for BPH are 5α-Reductase inhibitors and α-adrenergic blockers. These drugs, on the other hand, can have negative impacts on ejaculation, cognitive functions, and mental health [8,9]. Phytotherapies have emerged as an acceptable and additional option for BPH management because they are effective, less expensive, and well-tolerated [10].

The *Cordyceps* genus contains over 400 species, including *Cordyceps sinensis* and *Cordyceps militaris*, which have been utilized as a herbal and tonic medicine in China for thousands of years. They were used for a variety of health problems such as kidney and lung diseases and impotence [11]. Additionally, *Cordyceps militaris* has been widely utilized clinically for the management of chronic fatigue syndrome, chronic bronchitis, and diabetic nephropathy [11]. Cordycepin is an active compound found in the *Cordyceps militaris* caterpillar fungus [12]. Chemically, it is identical in structure to adenosine, however, cordycepin only lacks one 3′-hydroxyl group of the five-membered ribose ring [13]. Cordycepin has a wide range of pharmacological effects, the most studied of which are its antitumor [14], antioxidative [15], and anti-inflammatory properties as it suppresses the production of pro-inflammatory mediators in different cells [11]. Interestingly, it has been suggested that adenosine has antiproliferative activity via adenosine A3 receptor stimulation in several types of cancer [16,17]. Activation of this receptor was even found to inhibit prostate carcinoma cell growth [18]. Moreover, adenosine agonists have the potential to alleviate prostate smooth muscle contraction. Experimentally, electrical field stimulation of the contractile responses in isolated rat prostates was found to be attenuated by the activation of prejunctional A1 adenosine receptors [19]. Furthermore, α1 adrenoceptor-mediated responses of human prostatic stromal cells have been found to be inhibited by the stimulation of post-junctional A2A adenosine receptors [20]. Therefore, the current study was designed to assess the potential of cordycepin to attenuate BPH induced by testosterone in rats.

## 2. Materials and Methods

### 2.1. Chemicals

Cordycepin (>98%) was purchased from Salus Nutra Inc. (Xi’an, China). Testosterone enanthate was obtained as a kind gift from Chemical Industries Development Co. (CID), Giza, Egypt. Finasteride was obtained from Sigma-Aldrich (St. Louis, MO, USA). The chemicals used in this investigation were of the highest analytical quality.

### 2.2. Animals

Experimental work involving animals was conducted according to the approved procedures by the Faculty of Pharmacy’s Research Ethics Committee, King Abdulaziz University (Ref # PH-1443-23). Male Wistar rats (200–230 g) of 10-week of age were obtained from the animal facility of Faculty of Pharmacy, King Abdulaziz University. The rats were maintained under an air-conditioned environment (20–24 °C), alternating 12-h light/12-h dark cycles, and ad libitum feeding with a standard diet and water. The animals were acclimatized for 1 week to our experimental facility before the start of experiments.

### 2.3. Toxicity Study

To assess the acute toxicity of cordycepin based on the OECD Test Guideline 423, animals were administered an oral cordycepin dose of 2000 mg/kg. After that, the rats were observed closely during the first hour and regularly for the following 24 h of administration. As all animals survived, the experimental protocol was carried out again utilizing three additional rats. 

### 2.4. Experimental Design

Thirty-six male rats were separated into 6 groups randomly (6 animals per group). Group 1 (control) was subcutaneously (s.c.) administered normal saline (10 mL/kg, p.o.) and corn oil (1 mL/kg). Group 2 (cordycepin) was administered cordycepin dissolved in normal saline (10 mg/kg p.o.) and corn oil (s.c.). Group 3 (testosterone) was given normal saline (10 mL/kg, p.o.) and testosterone (3 mg/kg dissolved in corn oil, s.c.). Group 4 (testosterone + cordycepin 5 mg/kg) was administered cordycepin (5 mg/kg, p.o.) and testosterone (5 mg/kg s.c.). Group 5 (testosterone + cordycepin 10 mg/kg) was administered cordycepin (10 mg/kg, p.o.) and testosterone (5 mg/kg s.c.). Group 6 (positive control) was given finasteride (0.5 mg/kg p.o.) and testosterone (5 mg/kg s.c.). Administration of oral doses was carried out before s.c. injections by 1 h. Treatment of animals was administered once daily every 5 days per week for 4 consecutive weeks. All specified doses and treatment schedules were according to a pilot study. Prostate tissues from each animal group were harvested immediately after the sacrifice and 72 h after the last treatment. Parts of the prostatic ventral lobes were kept in a 10% neutral buffered formalin for histopathology and immunohistochemistry studies. The remaining tissues were flash-frozen in liquid nitrogen and stored at −80 °C for assessment of mRNA expression and biochemical markers.

### 2.5. Prostate Index and Weight

Prostate weights were registered after dissection, and hence the prostate index was calculated relative to the total body weight of the animal by dividing the prostate weight by the total body weight before multiplying by 1000. 

### 2.6. Histopathological Examination

Paraffin sections of 4 μm were obtained from the fixed prostate tissues. Following de-paraffinization and rehydration, the paraffin sections were stained with hematoxylin and eosin (H&E). The height of the prostate glandular epithelia was determined using Image J software (Image J, 1.46a, NIH, Bethesda, MD, USA).

### 2.7. Oxidative Status Markers Assessment

Phosphate-buffered saline containing 50 mM potassium phosphate (ice-cooled, pH 7.5) was used for the homogenization of prostatic tissues to determine malondialdehyde (MDA), glutathione (GSH), and superoxide dismutase (SOD) levels (Cat # 10009055, 703002 and 707002, Cayman Chemical, Ann Arbor, MI, USA, respectively). Total protein was assessed by a BCA protein assay kit (Cat # 355526, MyBioSource, Inc., San Diego, CA, USA).

### 2.8. Immunohistochemical Analyses

Tissue sections were deparaffinized, rehydrated with ethanol, and boiled in citrate buffer (pH 6.0) for 10 min. After a two-hour incubation in tris buffered saline (TBS) with 5% bovine serum albumin (BSA), the sections were immersed overnight at 4 °C with the following primary antibodies: rabbit monoclonal anti-cyclin D1 (Cat # ab16663), proliferating cell nuclear antigen (PCNA, Cat # ab92552), polyclonal anti-IL-6 (Cat # ab9324), monoclonal anti-TNF-α (Cat # ab220210), and polyclonal anti-NFκB (p65, Cat # ab194726, Cambridge, UK). After that, the slides were flushed with TBS and the primary antibodies were detected using Mouse- and Rabbit Specific-HRP/DAB (ABC) Detection IHC kit according to the manufacturer instructions (Cat # ab64264, ABCAM, Cambridge, UK). Image analysis of at least 3 sections per rat was conducted using Image J analysis software (Image J, 1.46a, NIH, Bethesda, MD, USA).

### 2.9. Analysis of mRNA Expression of Bax and Bcl2

An ultrasonic probe was used for homogenization of prostate tissues before extracting RNA using NucleoSpin RNA Mini kit (Macherey-Nagel GmbH & Co. KG, Duerin, Germany). The purity (A260/A280 ratio) and concentration of the extracted RNA was assessed using a spectrophotometer (Beckman, Brea, CA, USA) before carrying out the reverse transcription step using High-Capacity cDNA Reverse Transcription Kit (Cat # 4368814, Applied Biosystems, Foster City, CA, USA). For real-time polymerase chain reaction (qPCR), SYBR™ Green PCR Master Mix kit (Cat # 4309155, Applied Biosystems, Foster City, CA, USA) was utilized with the following forward primers Bax, Bcl2, and β-actin: 5′CCTGAGCTGACCTTGGAGCA, 5′GATAACCGGGAGATCGTGA, and 5′TCCGTCGCCGGTCCACACCC, respectively [21]. The reverse primers for Bax, Bcl2, and β-actin: 5′GGTGGTTGCCCTTTTCTACT, 5′AAAGCACATCCAATAAAAAGC, and 5′TCACCAACTGGGACGATATG, respectively [21]. Data analysis was according to the ΔΔCT method in which the data were normalized to β-actin readings [22]. 

### 2.10. Protein Expression of Bax, Bcl2, Total & Phosphor-AKT, and Total and Phosphor-AMPK by Western Blot

The prostates were homogenized by incubation in ice-cold radioimmunoprecipitation assay (RIPA) buffer for 30 min containing phosphatase and protease inhibitor cocktails. Following centrifugation at 3000 rpm for 30 min at 4 °C, the supernatant was used for the assay of protein content by a BCA protein assay kit (Cat # 355526, MyBioSource, Inc., San Diego, CA, USA). The protein lysates (80 µg/lane) were separated by 10% sodium dodecyl sulfate-polyacrylamide gel electrophoresis. After the electrophoresis step, the separated proteins were transferred onto an activated polyvinylidene difluoride membrane (Cat # ab133411, Abcam, Cambridge, UK). The membranes were blocked by 5% non-fat milk in TBST for 1 h at room temperature before overnight incubation with the following primary antibodies anti-Bax antibody (Cat # sc-23959) and anti-Bcl2 antibody (Cat # sc-7382) from Santa Cruz Biotechnology (Dallas, TX, USA), and anti-pan-AKT antibody (Cat # ab8805), anti-AKT (phospho T308) antibody (Cat # ab38449), anti-AMPK alpha antibody (Cat # ab32047), anti-AMPK alpha 1/2 (phospho T183/T172) antibody (Cat # ab133448), and anti-β actin antibody (Cat # ab6276) from Abcam (Cambridge, UK). After washing, the membranes were incubated for 1 hour at room temperature with HRP-conjugated secondary antibody (Cell Signaling Technology, Danvers, MA, USA) before membrane development using ECL Substrate kit (Cat # ab133406, Abcam, Cambridge, UK). Membrane visualization and protein quantification were performed using ChemiDoc MP Imaging System with Image Lab Software (Bio-Rad Laboratories, Dubai, United Arab Emirates) and Image J (1.46a, NIH, Bethesda, MD, USA), respectively. 

### 2.11. Statistical Analysis

Data are reported as mean ± SD. For statistical analysis with multiple comparisons using GraphPad Prism version 8.1. (GraphPad, La Jolla, CA, USA), one-way ANOVA with Tukey’s post-hoc test was utilized and a p-value of less than 0.05 is considered statistically significant. 

## 3. Results

### 3.1. Acute Toxicity Assessment

After 24 h of oral administration of cordycepin at 2000 mg/kg, there were no deaths detected among the three male rats utilised in this study. Hence, a following study was performed on three additional rats utilising the same experimental protocol, which also led to no deaths observed in the tested animals.

### 3.2. Prostate Weights and Indices

Principally, cordycepin at both doses tested was safe and well-tolerated as it resulted in no mortality or significant changes in prostate weights. In contrast, testosterone enanthate significantly increased prostate weight and index by 178.2% and 153.0%, respectively, relative to the control value (Table 1). However, coadministration with cordycepin at both 5 and 10 mg/kg led to a substantial decrease in prostate weight by 32.2% and 45.2% and prostate index by 29.5% and 41.5%, respectively, compared to rats that received testosterone alone. It was also noticeable that cotreatment of rats with finasteride at 0.5 mg/kg significantly decreased the prostate weight and index by 52.5% and 48.4% associated with testosterone administration.

### 3.3. Histopathological Examination

**Figure 1:**
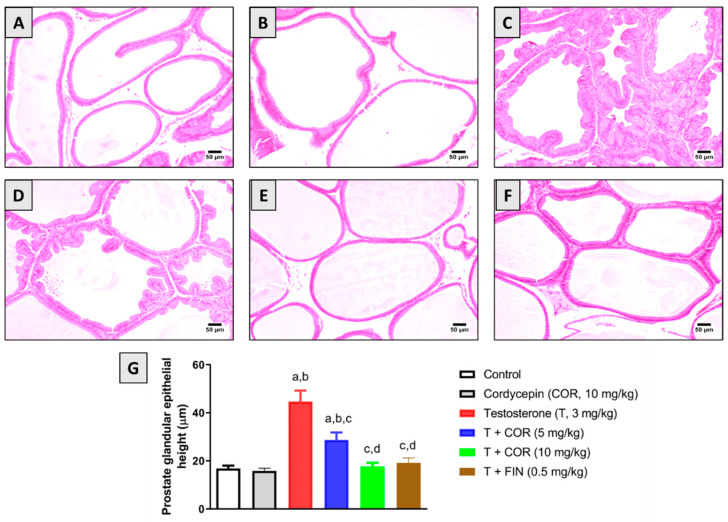
Effect of cordycepin on testosterone-induced pathological changes on prostate tissues: (**A**) control group and (**B**) cordycepin-alone group showing normal prostate tissue architecture; (**C**) testosterone-treated group (3 mg/kg) showing hyperplasia of prostatic acini; (**D**) cordycepin (5 mg/kg) + testosterone-treated group and (**E**) cordycepin (10 mg/kg) + testosterone-treated group showing moderate and high restoration of almost normal prostate histology, respectively; and (**F**) finasteride + testosterone group is without apparent acini hyperplasia. (**G**) Graphic presentation of prostate glandular epithelial height in the different treatment groups. Data are presented in the bar chart (*n* = 6) as mean ± SD. COR = cordycepin, T = testosterone, FIN = finasteride. a, b, c, or d: statistically different (*p* < 0.05) from control, cordycepin, testosterone or T + COR (5 mg/kg), respectively.

Microscopic examination of the prostate gland from the control and cordycepin-alone groups revealed normal histology of acini that were lined by cuboidal to low columnar epithelium with occasional papillary infoldings (Figure 1A,B). However, testosterone injection resulted in hyperplasia of prostatic acini that was characterized by frequent inward folding of the epithelial lining forming intraluminal projections. The hyperplastic cells appeared cuboidal to tall columnar with granular eosinophilic cytoplasm. Some examined sections showed expansion of the interstitial tissue with edema and inflammatory cell infiltration (Figure 1C). Regarding cordycepin (5 mg/kg)-treated animals, moderate improvement was detected in the examined sections as several acini showed hyperplasia of the epithelial lining accompanied by fewer areas of interstitial inflammatory regions (Figure 1D). The highest protection was observed in cordycepin (10 mg/kg)-treated animals as acinar epithelia appeared almost normal. Only fewer sections showed the epithelial lining hyperplasia with intraluminal projections (Figure 1E). The positive control group has obvious improvements against testosterone hyperplasia with almost normal prostatic architecture. Few acini showed desquamated epithelial cells into the acinar lumen admixed with eosinophilic tissue debris and inflammatory cells infiltration (Figure 1F).

### 3.4. Proliferation Markers

**Figure 2:**
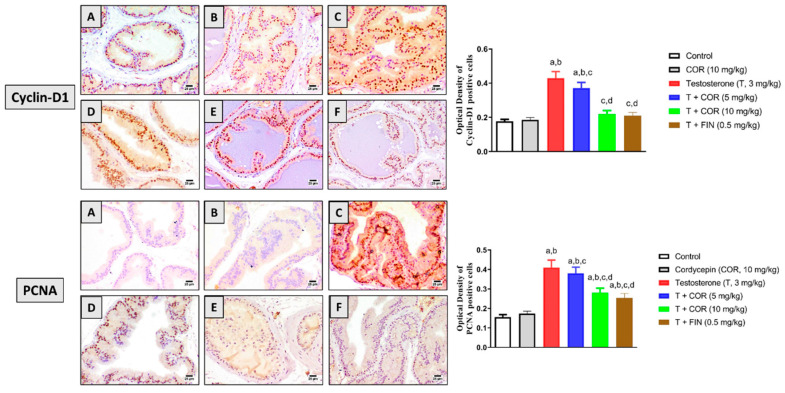
Effect of cordycepin on cyclin-D1 and PCNA expression as determined immunohistochemically in prostatic tissues of testosterone-treated rats. (**A**) Control, (**B**) cordycepin, (**C**) testosterone, (**D**) testosterone + cordycepin 5 mg/kg, (**E**) testosterone + cordycepin 10 mg/kg, and (**F**) testosterone + finasteride. Data presented in the bar charts (*n* = 6) are mean ± SD. COR = cordycepin, T = testosterone, FIN = finasteride. a, b, c, or d: statistically different (*p* < 0.05) from control, cordycepin, testosterone or T + COR (5 mg/kg), respectively.

Immunohistochemical analysis of the key regulators of cell proliferation cyclin-D1 and PCNA revealed an average number of positive cells for either of them in the control group (Figure 2A). Similarly, cordycepin resulted in no significant changes in the number of stained cells compared to the control group (Figure 2B). However, testosterone significantly induced a noticeable increase in the number of stained cells, indicating an elevated proliferation rate (Figure 2C). As shown in Figure 2D,E, the coadministration of cordycepin was capable of significantly ameliorating the substantial increment of cyclin D1 and PCNA-positive cells associated with administering testosterone alone. Interestingly, finasteride resulted in comparable findings to cordycepin at 10 mg/kg (Figure 2F). Densitometry data in Figure 2 revealed that cordycepin (10 mg/kg) significantly reduced the expression of cyclin D1 and PCNA by 48.8% and 31.7%, respectively.

### 3.5. Oxidative Stress Markers

The protective activity of cordycepin against oxidative stress induced by testosterone in prostatic tissues was also evaluated. Table 2 shows that testosterone exposure significantly elevated the levels of MDA, a lipid peroxidation marker, by around 3.5 folds while markedly depleting the content of GSH and reducing the activity of SOD by 64.0% and 71.4%, respectively, relative to the control values. Interestingly, treatment with cordycepin at 5 mg/kg and 10 mg/kg significantly attenuated the testosterone-induced increase in MDA levels by 33.0% and 42.1%. Moreover, cordycepin significantly ameliorated GSH depletion and SOD exhaustion by both doses tested. In a similar vein, finasteride was effective in ameliorating testosterone-induced oxidative stress as indicated by the MDA and GSH content and SOD activity levels.

### 3.6. Inflammatory Markers

**Figure 3:**
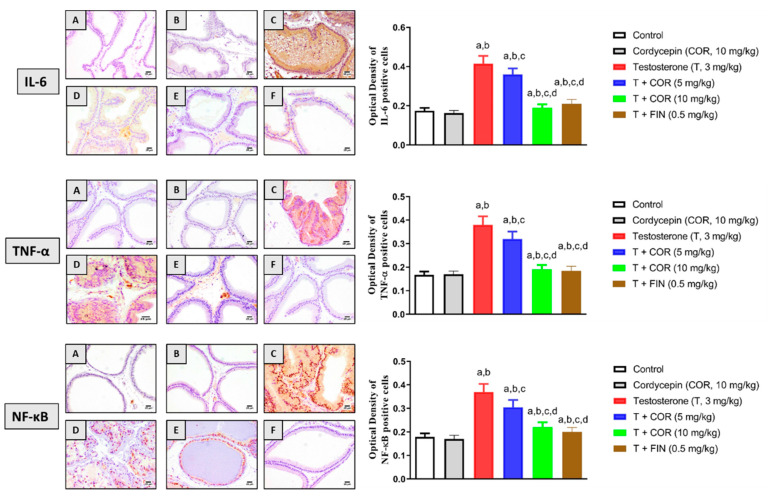
Effect of cordycepin on the expression of IL-6, TNF-α, and NF-κB as determined by immunohistochemistry in prostatic tissues of testosterone-treated rats. (**A**) Control, (**B**) cordycepin, (**C**) testosterone, (**D**) testosterone + cordycepin 5 mg/kg, (**E**) testosterone + cordycepin 10 mg/kg, and (**F**) testosterone + finasteride. Data (*n* = 6) presented in the bar charts are mean ± SD. COR = cordycepin, T = testosterone, FIN = finasteride. a, b, c, or d: statistically different from control, cordycepin, testosterone, or T + COR (5 mg/kg), respectively (*p* < 0.05).

Further immunohistochemical analysis was carried out to examine the inflammatory state of prostatic tissues in cordycepin-treated rats following testosterone administration (Figure 3). Testosterone significantly stimulated the expression of IL-6, while cordycepin treatment significantly ameliorated this increased expression of IL-6 by 13.2% and 54.2% at 5 mg/kg and 10 mg/kg, respectively. Furthermore, the expression of TNF-α and NF-κB were also induced with testosterone and these expression levels were markedly attenuated by cordycepin treatment at 5 mg/kg by 15.7% and 17.5%, respectively. Cordycepin at 10 mg/kg caused even further reduction in TNF-α expression by 49.7% and NF-κB expression by 40.5% compared to testosterone alone (Figure 3).

### 3.7. Expression of Bax and Bcl2

**Figure 4:**
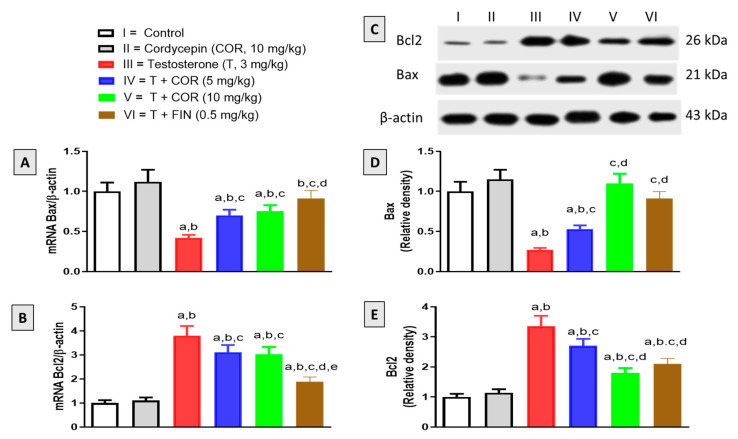
Cordycepin effect on prostatic expression of Bax (**A**) and Bcl2 (**B**) mRNAs and proteins (**C**) in testosterone-treated rats. Lanes I, II, III, IV, V, and VI represent control, cordycepin, testosterone, testosterone + cordycepin 5 mg/kg, testosterone + cordycepin 10 mg/kg, and testosterone + finasteride, respectively. Densitometric data of Bax and Bcl2 protein expression are demonstrated in bar charts (**D**,**E**), respectively. Data presented in the charts are mean ± SD. For mRNA and protein expression studies, *n* = 6 and 3, respectively. COR = cordycepin, T = testosterone, FIN = finasteride. a, b, c, d or e: significantly different (*p* < 0.05) from control, cordycepin, testosterone, T + COR (5 mg/kg) or T + COR (10 mg/kg), respectively.

Cordycepin’s antiapoptotic activity was examined according to Bax and Bcl2 mRNA and protein expression in prostatic tissues of testosterone-treated rats. As shown in Figure 4A,D, testosterone markedly decreased the mRNA and protein expression of the pro-apoptotic protein Bax, respectively. Yet, cordycepin significantly attenuated this decrease in the expression of Bax at all doses tested in this study. With regards to the antiapoptotic protein Bcl2, testosterone significantly increased its mRNA and protein expression, however, cordycepin significantly attenuated these changes at 5 mg/kg and 10 mg/kg (Figure 4B,E). Finasteride also showed significant anti-apoptotic activity, as indicated by the induced expression of Bax and the reduced expression of Bcl2 relative to the testosterone-alone group (Figure 4).

### 3.8. Expression of p-AKT and p-AMPK

**Figure 5:**
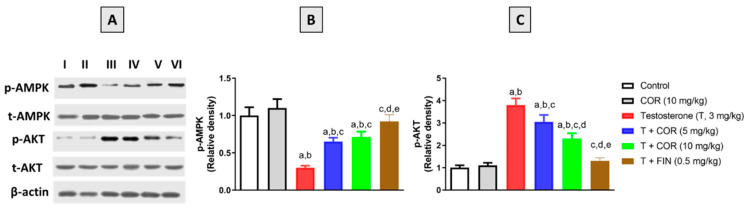
Effect of cordycepin on prostatic protein expression of p-AKT and p-AMPK (**A**) in testosterone-treated rats. Lanes I, II, III, IV, V, and VI represent control, cordycepin, testosterone, testosterone + cordycepin 5 mg/kg, testosterone + cordycepin 10 mg/kg, and testosterone + finasteride, respectively. Densitometric data of p-AKT and p-AMPK expression presented in bar charts (**B**,**C**) are mean ± SD (*n* = 6). COR = cordycepin, T = testosterone, FIN = finasteride. a, b, c, d or e: significantly different (*p* < 0.05) from control, cordycepin, testosterone, T + COR (5 mg/kg) or T + COR (10 mg/kg), respectively.

Finally, the phosphorylated and total protein forms of AMPK and AKT were analyzed to study the cellular targets driving the beneficial effects of cordycepin that were observed throughout this study. As can be seen in Figure 5A, testosterone treatment led to a noticeable decrease in p-AMPK concomitant with an increase in p-AKT expression. However, cordycepin treatment at doses of 5 mg/kg and 10 mg/kg administered to testosterone-challenged animals resulted in significant amelioration of p-AMPK inhibition as well as p-AKT enhanced expression. The 5-α reductase inhibitor finasteride also enhanced p-AMPK expression and inhibited p-AKT protein content associated with testosterone administration (Figure 5B,C).

## 4. Discussion

BPH is one of the most prevalent benign tumors in men, with increasing prevalence after 40 years of age [23]. Uncontrolled cell proliferation, oxidative stress, disturbed cell apoptosis, and inflammation participate in the development of BPH [24,25,26]. Symptoms of BPH such as lower urinary tract symptoms can negatively influence the quality of life of affected men. The main available approaches for pharmacological management of this disease involve using adrenergic α-1 antagonists and 5-α reductase inhibitors [27]. However, it is known that these pharmacological management options may cause intolerable side effects including postural hypotension, impotence, and gynecomastia, which can affect patient compliance [28,29]. Cordycepin is a nucleoside analog that is commonly isolated from the fermentative fluid and fruiting bodies of the entomopathogenic fungus *Cordyceps militaris* [30]. Cordycepin has several biological and pharmacological properties reported in the literature including the regulation of steroidogenesis, cell proliferation, inflammation, and apoptosis through various signaling pathways [31,32,33,34]. Hence, our study was conducted to investigate the potential of cordycepin to mitigate BPH induced by testosterone in rats.

In this study, it was observed that treatment with cordycepin for 4 weeks significantly attenuated increased prostate weights and indices associated with testosterone administration. Furthermore, histopathological examination confirmed these results as cordycepin cotreatment was associated with almost normal acinar epithelia. In accordance with these findings, it was reported that *Cordyceps militaris* fruiting body extract tended to inhibit the increase in prostate weight induced by the administration of testosterone over 30 days [35]. Our findings are also in line with the established antiproliferative activity of cordycepin against various cells, including those of the prostate [36,37]. This can be explained by the ability of cordycepin to activate the A3 adenosine receptor, and its activation is associated with the inhibition of prostate cancer cell proliferation [16,17,18]. 

Several studies also highlight a major role of oxidative stress in the pathogenesis of testosterone-induced BPH [38]. Interestingly, our findings demonstrated that cordycepin possesses significant antioxidant activity as evidenced by the reduced lipid peroxidation and antioxidant enzyme exhaustion. This is in harmony with previous findings reporting the significant antioxidant activity of cordycepin [39,40,41]. In addition, these results are parallel with several reports correlating the anti-oxidation of many compounds with protection against PBH [42,43,44].

Accumulating evidence indicates a significant role of inflammation in the development of testosterone-induced BPH [45]. In this study, cordycepin demonstrated significant anti-inflammatory activity supported by the decreased expression of IL-6, TNF-α, and NF-κB in the prostatic tissues of testosterone-challenged rats. These findings gain support from previous reports highlighting a significant anti-inflammatory activity of cordycepin mediated through suppression of NF-κB signaling pathways [41,46,47,48]. Oxidative stress can trigger certain inflammatory pathways via activating transcription factors including NF-κB, which in turn control the gene expression of proinflammatory mediators such as TNF-α [49,50,51]. Hence, the decreased expression of inflammatory mediators associated with cordycepin could be partly due to its considerable antioxidant activity. 

Prostatic inflammation can lead to stromal and glandular hyperplasia by disturbing the balance between cell proliferation and cell death in the prostate [52]. In the same vein, testosterone is known to induce pro-proliferative and anti-apoptotic changes in cycline-D1, PCNA, Bax, and Bcl2 mRNA content in the prostate gland [8]. In this study, cordycepin at both tested doses significantly ameliorated these pro-proliferative and anti-apoptotic changes induced by testosterone administration. These findings are in harmony with those in the literature describing the antiproliferative activity of cordycepin against prostate cells and other types of cells that involve regulating the expression of cyclin-D1, PCNA, Bax, and Bcl2 [37,53,54,55,56]. 

**Figure 6:**
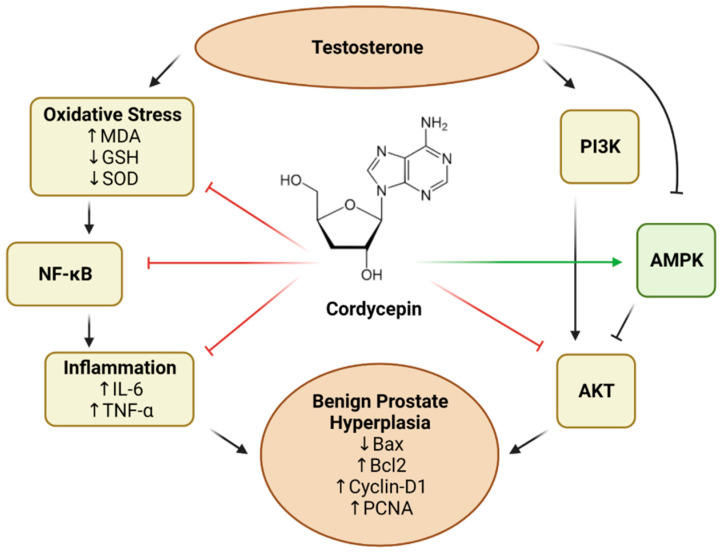
Graphical representation of the proposed mechanism of protection offered by cordycepin against BPH induced by testosterone. Blunt-head arrows indicate inhibition, whereas pointed-head arrows reflect stimulation.

Interestingly, cordycepin was noted to inhibit cell proliferation by modulating AKT signaling in several types of cancer, including prostate cancer [57,58,59,60,61]. It is known that AKT signaling plays a significant role in BPH development as its overexpression is associated with an increased prostate size [62,63]. AKT signaling activation is also associated with the dysregulation of cell death by reducing the expression of Bax and inducing the expression of Bcl-2 [64]. Hence, the potential of cordycepin to inhibit AKT activity in BPH was explored in this study. It was found that testosterone alone significantly induced the expression levels of p-AKT in rat prostate. This is in line with reported findings that PI3K/AKT activation is essential for cell proliferation in testosterone-induced BPH [65]. Remarkably, cordycepin in the current study significantly attenuated this rise in p-AKT expression in the prostatic tissues of testosterone-treated rats. Moreover, cordycepin caused significant amelioration of testosterone-induced decrease in the prostatic expression of p-AMPK. Similar findings were reported in several studies where cordycepin has been shown to activate AMPK and inhibit AKT activation [58,59,60,61]. In this regard, it has been demonstrated that BPH involves lower levels of p-AMPK, and its activation has been proposed to counter-modulate the signaling of pro-inflammatory mediators [66,67,68,69]. This is also in harmony with previous findings demonstrating the beneficial effects of targeting the PI3K/AKT pathway in BPH [63,70,71,72]. The suggested mechanism of actions of cordycepin are illustrated in Figure 6. 

## 5. Conclusions

Overall, cordycepin offers protection against testosterone-induced BPH in rats. This protective activity can be at least partially explained by its antiproliferative, antioxidant, anti-inflammatory, and pro-apoptotic actions as well as its modulation of AMPK and AKT activation.

## Figures and Tables

**Table 1:** Effect of cordycepin on prostate weight and index in testosterone-treated rats.

	Final Body Weight (g)	Prostate Weight (mg)	Prostate Index (×10^3^)
Control	245 ± 20.2	281 ± 30.1	1.15 ± 0.16
Cordycepin (COR 10 mg/kg)	250 ± 21.2	305 ± 32.5	1.23 ± 0.19
Testosterone (T; 3 mg/kg)	272 ± 30.8	782 ^a,b^ ± 81.4	2.91 ^a,b^ ± 0.48
T + COR (5 mg/kg)	260 ± 22.8	530 ^a,b,c^ ± 58.3	2.05 ^a,b,c^ ± 0.26
T + COR (10 mg/kg)	255 ± 26.2	428 ^a,b,c,d^ ± 46.7	1.70 ^a,c^ ± 0.31
T + FIN (0.5 mg/kg)	251 ± 27.2	371 ^a,c,d^ ± 32.5	1.50 ^c,d^ ± 0.23

Data (*n* = 6) are expressed as mean ± SD. COR = cordycepin, T = testosterone, FIN = finasteride. a, b, c, or d: statistically different (*p* < 0.05) from control, cordycepin, testosterone or T + COR (5 mg/kg), respectively.

**Table 2:** Effect of cordycepin on oxidative status in prostatic tissues of testosterone-treated rats.

	MDA (nmol/mg Protein)	GSH (mmol/mg Protein)	SOD (U/mg Protein)
Control	18.77 ± 2.10	89.00 ± 9.73	8.3 ± 0.91
Cordycepin (COR 10 mg/kg)	17.11 ± 1.86	97.41 ± 10.2	8.8 ± 0.98
Testosterone (T; 3 mg/kg)	65.10 ^a,b^ ± 7.53	32.17 ^a,b^ ± 3.41	2.37 ^a,b^ ± 0.30
T + COR (5 mg/kg)	43.61 ^a,b,c^ ± 4.51	66.31 ^a,b,c^ ± 7.05	6.53 ^a,c,b^ ± 0.68
T + COR (10 mg/kg)	37.71 ^a,b,c^ ± 4.05	75.52 ^b,c^ ± 6.34	6.62 ^a,b,c^ ± 0.72
T + FIN (0.5 mg/kg)	27.52 ^a,b,c,d,e^ ± 2.3	80.45 ^b,c,d^ ± 8.7	8.6 ^c,d,e^ ± 0.96

Data (*n* = 6) are expressed as mean ± SD. COR = cordycepin, T = testosterone, FIN = finasteride. a, b, c, d or e: statistically different (*p* < 0.05) from control, cordycepin, testosterone, T + COR (5 mg/kg) or T + COR (10 mg/kg), respectively.

## Data Availability

The data presented in this study are available on request from the corresponding author.

## References

[1] Chughtai B., Lee R., Te A., Kaplan S. (2011). Role of inflammation in benign prostatic hyperplasia. Rev. Urol..

[2] Patel N.D., Parsons J.K. (2014). Epidemiology and etiology of benign prostatic hyperplasia and bladder outlet obstruction. Indian J. Urol..

[3] Bellinger A.S., Elliott S.P., Yang L., Wei J.T., Saigal C.S., Smith A., Wilt T.J., Strope S.A., Project T.U.D.I.A. (2012). Changes in initial expenditures for benign prostatic hyperplasia evaluation in the Medicare population: A comparison to overall Medicare inflation. J. Urol..

[4] Ventura S., Oliver V.L., White C.W., Xie J.H., Haynes J.M., Exintaris B. (2011). Novel drug targets for the pharmacotherapy of benign prostatic hyperplasia (BPH). Br. J. Pharmacol..

[5] Devlin C.M., Simms M.S., Maitland N.J. (2021). Benign prostatic hyperplasia–what do we know?. BJU Int..

[6] Bushman W.A., Jerde T.J. (2016). The role of prostate inflammation and fibrosis in lower urinary tract symptoms. Am. J. Physiol. Physiol..

[7] Minciullo P.L., Inferrera A., Navarra M., Calapai G., Magno C., Gangemi S. (2015). Oxidative stress in benign prostatic hyperplasia: A systematic review. Urol. Int..

[8] Eid B.G., Abdel-Naim A.B. (2020). Piceatannol attenuates testosterone-induced benign prostatic hyperplasia in rats by modulation of Nrf2/HO-1/NFκB axis. Front. Pharmacol..

[9] Kim J.H., Baek M.J., Sun H.Y., Lee B., Li S., Khandwala Y., Del Giudice F., Chung B.I. (2018). Efficacy and safety of 5 alpha-reductase inhibitor monotherapy in patients with benign prostatic hyperplasia: A meta-analysis. PLoS ONE.

[10] Csikós E., Horváth A., Ács K., Papp N., Balázs V.L., Dolenc M.S., Kenda M., Glavač N.K., Nagy M., Protti M. (2021). Treatment of benign prostatic hyperplasia by natural drugs. Molecules.

[11] Tan L., Song X., Ren Y., Wang M., Guo C., Guo D., Gu Y., Li Y., Cao Z., Deng Y. (2021). Anti-inflammatory effects of cordycepin: A review. Phyther. Res..

[12] Cunningham K.G., Manson W., Spring F.S., Hutchinson S.A. (1950). Cordycepin, a metabolic product isolated from cultures of *Cordyceps militaris* (Linn.) Link. Nature.

[13] Jeong J.-W., Jin C.-Y., Park C., Hong S.H., Kim G.-Y., Jeong Y.K., Lee J.-D., Yoo Y.H., Choi Y.H. (2011). Induction of apoptosis by cordycepin via reactive oxygen species generation in human leukemia cells. Toxicol. Vitr..

[14] Nakamura K., Shinozuka K., Yoshikawa N. (2015). Anticancer and antimetastatic effects of cordycepin, an active component of Cordyceps sinensis. J. Pharmacol. Sci..

[15] Ramesh T., Yoo S.-K., Kim S.-W., Hwang S.-Y., Sohn S.-H., Kim I.-W., Kim S.-K. (2012). Cordycepin (3′-deoxyadenosine) attenuates age-related oxidative stress and ameliorates antioxidant capacity in rats. Exp. Gerontol..

[16] Cao H.-L., Liu Z.-J., Chang Z. (2017). Cordycepin induces apoptosis in human bladder cancer cells via activation of A3 adenosine receptors. Tumor Biol..

[17] Nakamura K., Yoshikawa N., Yamaguchi Y.U., Kagota S., Shinozuka K., Kunitomo M. (2006). Antitumor effect of cordycepin (3′-deoxyadenosine) on mouse melanoma and lung carcinoma cells involves adenosine A3 receptor stimulation. Anticancer Res..

[18] Fishman P., Bar-Yehuda S., Ardon E., Rath-Wolfson L., Barrer F., Ochaion A., Madi L. (2003). Targeting the A3 adenosine receptor for cancer therapy: Inhibition of prostate carcinoma cell growth by A3AR agonist. Anticancer Res..

[19] Preston A., Lau W.A.K., Pennefather J.N., Ventura S. (2000). Effects of adenine nucleosides and nucleotides on neuromuscular transmission to the prostatic stroma of the rat. Br. J. Pharmacol..

[20] Preston A., Frydenberg M., Haynes J.M. (2004). A1 and A2A adenosine receptor modulation of α1-adrenoceptor-mediated contractility in human cultured prostatic stromal cells. Br. J. Pharmacol..

[21] Sirwi A., Shaik R.A., Alamoudi A.J., Eid B.G., Kammoun A.K., Ibrahim S.R., Mohamed G.A., Abdallah H.M., Abdel-Naim A.B. (2021). Mokko lactone attenuates doxorubicin-induced hepatotoxicity in rats: Emphasis on Sirt-1/FOXO1/NF-κB axis. Nutrients.

[22] Livak K.J., Schmittgen T.D. (2001). Analysis of relative gene expression data using real-time quantitative PCR and the 2^−ΔΔCT^ method. Methods.

[23] Donnell R.F. (2011). Benign prostate hyperplasia: A review of the year’s progress from bench to clinic. Curr. Opin. Urol..

[24] Pawlicki B., Zieliński H., Dabrowski M. (2004). Role of apoptosis and chronic prostatitis in the pathogenesis of benign prostatic hyperplasia. Pol. Merkur. Lek. Organ Pol. Tow. Lek..

[25] Aaron L., Franco O.E., Hayward S.W. (2016). Review of prostate anatomy and embryology and the etiology of benign prostatic hyperplasia. Urol. Clin..

[26] Udensi U.K., Tchounwou P.B. (2016). Oxidative stress in prostate hyperplasia and carcinogenesis. J. Exp. Clin. Cancer Res..

[27] Silva J., Silva C.M., Cruz F. (2014). Current medical treatment of lower urinary tract symptoms/BPH: Do we have a standard?. Curr. Opin. Urol..

[28] Traish A.M., Hassani J., Guay A.T., Zitzmann M., Hansen M.L. (2011). Adverse side effects of 5α-reductase inhibitors therapy: Persistent diminished libido and erectile dysfunction and depression in a subset of patients. J. Sex. Med..

[29] Nickel J.C., Sander S., Moon T.D. (2008). A meta-analysis of the vascular-related safety profile and efficacy of α-adrenergic blockers for symptoms related to benign prostatic hyperplasia. Int. J. Clin. Pract..

[30] Quy T.N., Xuan T.D., Andriana Y., Tran H.-D., Khanh T.D., Teschke R. (2019). Cordycepin isolated from *Cordyceps militaris*: Its newly discovered herbicidal property and potential plant-based novel alternative to glyphosate. Molecules.

[31] Leu S.-F., Poon S.L., Pao H.-Y., Huang B.-M. (2011). The in vivo and in vitro stimulatory effects of cordycepin on mouse leydig cell steroidogenesis. Biosci. Biotechnol. Biochem..

[32] Jeong J.-W., Jin C.-Y., Kim G.-Y., Lee J.-D., Park C., Kim G.-D., Kim W.-J., Jung W.-K., Kil Seo S., Choi I.-W. (2010). Anti-inflammatory effects of cordycepin via suppression of inflammatory mediators in BV2 microglial cells. Int. Immunopharmacol..

[33] Baik J.-S., Mun S.-W., Kim K.-S., Park S.-J., Yoon H.-K., Kim D.-H., Park M.-K., Kim C.-H., Lee Y.-C. (2016). Apoptotic effects of cordycepin through the extrinsic pathway and p38 MAPK activation in human glioblastoma U87MG cells. J. Microbiol. Biotechnol..

[34] Pan B.-S., Wang Y.-K., Lai M.-S., Mu Y.-F., Huang B.-M. (2015). Cordycepin induced MA-10 mouse Leydig tumor cell apoptosis by regulating p38 MAPKs and PI3K/AKT signaling pathways. Sci. Rep..

[35] Kusama K., Miyagawa M., Ota K., Kuwabara N., Saeki K., Ohnishi Y., Kumaki Y., Aizawa T., Nakasone T., Okamatsu S. (2020). *Cordyceps militaris* fruit body extract decreases testosterone catabolism and testosterone-stimulated prostate hypertrophy. Nutrients.

[36] Yoon S.Y., Park S.J., Park Y.J. (2018). The anticancer properties of cordycepin and their underlying mechanisms. Int. J. Mol. Sci..

[37] Lee H.H., Park C., Jeong J.-W., Kim M.J., Seo M.J., Kang B.W., Park J.U., Kim G.-Y., Choi B.T., Choi Y.H. (2013). Apoptosis induction of human prostate carcinoma cells by cordycepin through reactive oxygen species-mediated mitochondrial death pathway. Int. J. Oncol..

[38] Vital P., Castro P., Ittmann M. (2016). Oxidative stress promotes benign prostatic hyperplasia. Prostate.

[39] Wang F., Yin P., Lu Y., Zhou Z., Jiang C., Liu Y., Yu X. (2015). Cordycepin prevents oxidative stress-induced inhibition of osteogenesis. Oncotarget.

[40] Park J.M., Lee J.S., Lee K.R., Ha S.-J., Hong E.K. (2014). *Cordyceps militaris* extract protects human dermal fibroblasts against oxidative stress-induced apoptosis and premature senescence. Nutrients.

[41] Han F., Dou M., Wang Y., Xu C., Li Y., Ding X., Xue W., Zheng J., Tian P., Ding C. (2020). Cordycepin protects renal ischemia/reperfusion injury through regulating inflammation, apoptosis, and oxidative stress. Acta Biochim. Biophys. Sin..

[42] Ammar A.E., Esmat A., Hassona M.D.H., Tadros M.G., Abdel-Naim A.B., Guns E.S.T. (2015). The effect of pomegranate fruit extract on testosterone-induced BPH in rats. Prostate.

[43] Atawia R.T., Tadros M.G., Khalifa A.E., Mosli H.A., Abdel-Naim A.B. (2013). Role of the phytoestrogenic, pro-apoptotic and anti-oxidative properties of silymarin in inhibiting experimental benign prostatic hyperplasia in rats. Toxicol. Lett..

[44] Wu X., Gu Y., Li L. (2017). The anti-hyperplasia, anti-oxidative and anti-inflammatory properties of Qing Ye Dan and swertiamarin in testosterone-induced benign prostatic hyperplasia in rats. Toxicol. Lett..

[45] Rastrelli G., Vignozzi L., Corona G., Maggi M. (2019). Testosterone and benign prostatic hyperplasia. Sex. Med. Rev..

[46] Choi Y.H., Kim G.-Y., Lee H.H. (2014). Anti-inflammatory effects of cordycepin in lipopolysaccharide-stimulated RAW 264.7 macrophages through Toll-like receptor 4-mediated suppression of mitogen-activated protein kinases and NF-κB signaling pathways. Drug Des. Devel. Ther..

[47] Ashraf S., Radhi M., Gowler P., Burston J.J., Gandhi R.D., Thorn G., Piccinini A.M., Walsh D., Chapman V., De Moor C.H. (2019). The polyadenylation inhibitor cordycepin reduces pain, inflammation and joint pathology in rodent models of osteoarthritis. Sci. Rep..

[48] Hwang I.-H., Oh S.Y., Jang H.-J., Jo E., Joo J.C., Lee K.-B., Yoo H.-S., Lee M.Y., Park S.J., Jang I.-S. (2017). Cordycepin promotes apoptosis in renal carcinoma cells by activating the MKK7-JNK signaling pathway through inhibition of c-FLIPL expression. PLoS ONE.

[49] Rahman I. (2003). Oxidative stress, chromatin remodeling and gene transcription in inflammation and chronic lung diseases. BMB Rep..

[50] Hoffmann A., Natoli G., Ghosh G. (2006). Transcriptional regulation via the NF-κB signaling module. Oncogene.

[51] Elsharkawy A.M., Mann D.A. (2007). Nuclear factor-κB and the hepatic inflammation-fibrosis-cancer axis. Hepatology.

[52] Chughtai B., Forde J.C., Thomas D.D.M., Laor L., Hossack T., Woo H.H., Te A.E., Kaplan S.A. (2016). Benign prostatic hyperplasia. Nat. Rev. Dis. Prim..

[53] Lee H.H., Kim S.O., Kim G.-Y., Moon S.-K., Kim W.-J., Jeong Y.K., Yoo Y.H., Choi Y.H. (2014). Involvement of autophagy in cordycepin-induced apoptosis in human prostate carcinoma LNCaP cells. Environ. Toxicol. Pharmacol..

[54] Chang M.-M., Hong S.-Y., Yang S.-H., Wu C.-C., Wang C.-Y., Huang B.-M. (2020). Anti-cancer effect of cordycepin on FGF9-induced testicular tumorigenesis. Int. J. Mol. Sci..

[55] Yoshikawa N., Yamada S., Takeuchi C., Kagota S., Shinozuka K., Kunitomo M., Nakamura K. (2008). Cordycepin (3′-deoxyadenosine) inhibits the growth of B16-BL6 mouse melanoma cells through the stimulation of adenosine A3 receptor followed by glycogen synthase kinase-3β activation and cyclin D1 suppression. Naunyn. Schmiedebergs. Arch. Pharmacol..

[56] Jang H.J., Yang K.E., Hwang I.H., Huh Y.H., Kim D.J., Yoo H.S., Park S.J., Jang I.S. (2019). Cordycepin inhibits human ovarian cancer by inducing autophagy and apoptosis through Dickkopf-related protein 1/β-catenin signaling. Am. J. Transl. Res..

[57] Lee J.-D., Jeong J.-W., Jin C.-Y., Park C., Han M.H., Kim G.-Y., Moon S.-K., Gil Kim C., Jeong Y.K., Kim W.-J. (2012). Inhibition of migration and invasion of LNCaP human prostate carcinoma cells by cordycepin through inactivation of Akt. Int. J. Oncol..

[58] Li H.-B., Chen J.-K., Su Z.-X., Jin Q.-L., Deng L.-W., Huang G., Shen J.-N. (2021). Cordycepin augments the chemosensitivity of osteosarcoma to cisplatin by activating AMPK and suppressing the AKT signaling pathway. Cancer Cell Int..

[59] Liao X.-Z., Gao Y., Zhao H.-W., Zhou M., Chen D.-L., Tao L.-T., Guo W., Sun L.-L., Gu C.-Y., Chen H.-R. (2021). Cordycepin reverses cisplatin resistance in non-small cell lung cancer by activating AMPK and inhibiting AKT signaling pathway. Front. Cell Dev. Biol..

[60] Gao Y., Chen D.-L., Zhou M., Zheng Z.-S., He M.-F., Huang S., Liao X.-Z., Zhang J.-X. (2020). Cordycepin enhances the chemosensitivity of esophageal cancer cells to cisplatin by inducing the activation of AMPK and suppressing the AKT signaling pathway. Cell Death Dis..

[61] Bi Y., Li H., Yi D., Sun Y., Bai Y., Zhong S., Song Y., Zhao G., Chen Y. (2018). Cordycepin augments the chemosensitivity of human glioma cells to temozolomide by activating AMPK and inhibiting the AKT signaling pathway. Mol. Pharm..

[62] Sreenivasulu K., Nandeesha H., Dorairajan L.N., Ganesh R.N. (2018). Over expression of PI3K-AkT reduces apoptosis and increases prostate size in benign prostatic hyperplasia. Aging Male.

[63] Jin P., Wang Y.-H., Peng Y.-G., Hu S., Lu Q., Yang L.-Y. (2010). Effect of PI3K/AKT inhibitor on benign prostate hyperplasia and its mechanism: An experimental study. Natl. J. Androl..

[64] Caggia S., Libra M., Malaponte G., Cardile V. (2011). Modulation of YY1 and p53 expression by transforming growth factor-β3 in prostate cell lines. Cytokine.

[65] Cha J.Y., Wee J., Jung J., Jang Y., Lee B., Hong G.-S., Chang B.C., Choi Y.-L., Shin Y.K., Min H.-Y. (2015). Anoctamin 1 (TMEM16A) is essential for testosterone-induced prostate hyperplasia. Proc. Natl. Acad. Sci. USA.

[66] Vanella L., Russo G.I., Cimino S., Fragalà E., Favilla V., Volti G.L., Barbagallo I., Sorrenti V., Morgia G. (2014). Correlation between lipid profile and heme oxygenase system in patients with benign prostatic hyperplasia. Urology.

[67] Green C.J., Macrae K., Fogarty S., Hardie D.G., Sakamoto K., Hundal H.S. (2011). Counter-modulation of fatty acid-induced pro-inflammatory nuclear factor κB signalling in rat skeletal muscle cells by AMP-activated protein kinase. Biochem. J..

[68] Lim R., Barker G., Lappas M. (2015). Activation of AMPK in human fetal membranes alleviates infection-induced expression of pro-inflammatory and pro-labour mediators. Placenta.

[69] Zhang M., Wang S., Pan Z., Ou T., Ma J., Liu H., Li R., Yang P., Han W., Guan S. (2018). AMPK/NF-κB signaling pathway regulated by ghrelin participates in the regulation of HUVEC and THP1 Inflammation. Mol. Cell. Biochem..

[70] Kortam M.A., Alawady A.S., Sadik N.A.H., Fathy N. (2022). Fenofibrate mitigates testosterone induced benign prostatic hyperplasia via regulation of Akt/FOXO3a pathway and modulation of apoptosis and proliferation in rats. Arch. Biochem. Biophys..

[71] Wang S., Li K., Liu Z., Gui S., Liu N., Liu X. (2021). Aerobic exercise ameliorates benign prostatic hyperplasia in obese mice through downregulating the AR/androgen/PI3K/AKT signaling pathway. Exp. Gerontol..

[72] Mosli H.H., Esmat A., Atawia R.T., Shoieb S.M., Mosli H.A., Abdel-Naim A.B. (2015). Metformin attenuates testosterone-induced prostatic hyperplasia in rats: A pharmacological perspective. Sci. Rep..

